# Zepyros: a webserver to evaluate the shape complementarity of protein–protein interfaces

**DOI:** 10.1093/bioadv/vbaf051

**Published:** 2025-03-20

**Authors:** Mattia Miotto, Lorenzo Di Rienzo, Leonardo Bo’, Giancarlo Ruocco, Edoardo Milanetti

**Affiliations:** Center for Life Nano & Neuroscience, Italian Institute of Technology, Rome 00161, Italy; Center for Life Nano & Neuroscience, Italian Institute of Technology, Rome 00161, Italy; Center for Life Nano & Neuroscience, Italian Institute of Technology, Rome 00161, Italy; Center for Life Nano & Neuroscience, Italian Institute of Technology, Rome 00161, Italy; Department of Physics, Sapienza University of Rome, Rome 00185, Italy; Center for Life Nano & Neuroscience, Italian Institute of Technology, Rome 00161, Italy; Department of Physics, Sapienza University of Rome, Rome 00185, Italy

## Abstract

**Motivation:**

Shape complementarity of molecular surfaces at the interfaces is a well-known characteristic of protein–protein binding regions, and it is critical in influencing the stability of the complex. Measuring such complementarity is of great importance for a number of theoretical and practical implications; however, only a limited number of tools are currently available to efficiently and rapidly assess it.

**Results:**

Here, we introduce Zepyros (ZErnike Polynomials analYsis of pROtein Shapes), a webserver for fast measurement of the shape complementarity between two molecular interfaces of a given protein–protein complex using structural information. Zepyros is implemented as a publicly available tool with a user-friendly interface.

**Availability and implementation:**

Our server can be found at the following link (all major browser supported): https://zepyros.bio-groups.com.

## 1 Introduction

Knowing the stability of protein–protein complexes has important theoretical implications that are intimately linked to organization of the residue side-chains of the protein partners binding regions (Hadi-Alijanvand 2020, Desantis *et al.* 2022, Miotto *et al.* 2022, 2023), the characterization of which provides information on the nature of the binding of the interacting proteins, and a wide range of potential applications from drug design to the optimization of antibody activity (Manhart and Morozov 2015, Norman *et al.* 2020, Di Rienzo *et al.* 2021, De Lauro *et al.* 2022).

Although it is not trivial to define the direct correspondence between the physico-chemical properties of the interfaces and the stability of the protein complex, it is widely known that among the different factors involved in the stabilization of protein complexes, the contribution of the van der Waals forces plays a key role. The minimization of the energy contribution of the van der Waals interactions is due to the rearrangement of the side chains of the residues at the interface, thus determining the optimization of the shape complementarity between the molecular surfaces.

Indeed, increasing evidence shows that local shape complementarity is not only a necessary feature for the identification of binding regions, but also to determine the stability of the resulting complex in contrast with electrostatic compatibility, which seems to play a more subtle effect (Li *et al.* 2013, Grassmann *et al.* 2023).

Based on these observations, we previously proposed the innovative approach of representing protein molecular surface in terms of the sum of 2D Zernike polynomials so that comparing the decomposition coefficients between couples of surface patches provides a compact and rapid way to assess shape complementarity (Milanetti *et al.* 2021, Grassmann *et al.* 2023). Our parameter-free method allows to distinguish between binding regions and random portions of the protein molecular surfaces with an accuracy of ∼72% and area under the receiver operating characteristic curve of 80% (see Section 2). Here, we present Zepyros (ZErnike Polynomials analYsis of pROtein Shapes), a free web application that allows for a rapid evaluation of shape complementarity at the interface of bounded protein complexes.

## 2 Materials and methods

**Figure 1.**
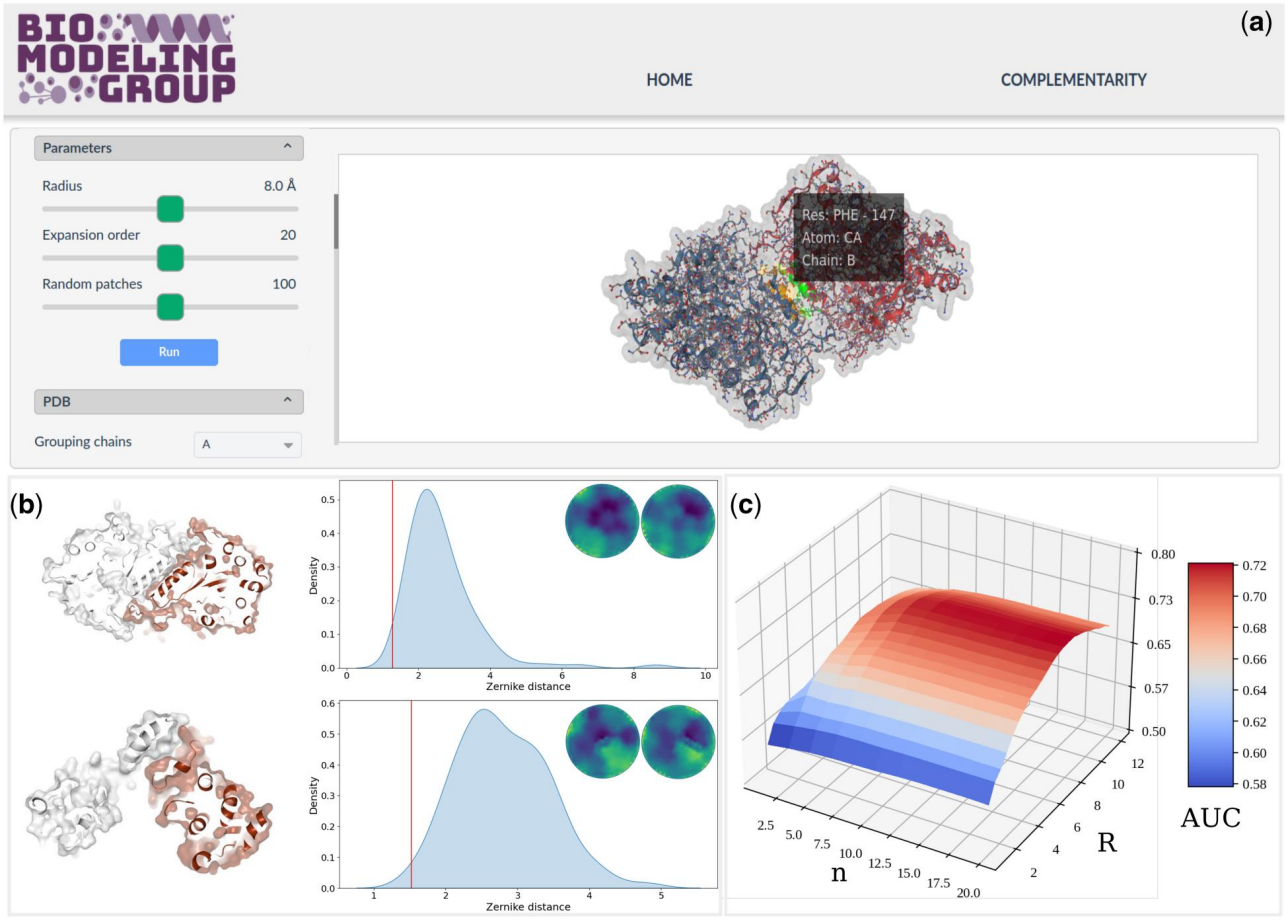
(a) Input page of the Zepyros server (running examples are provided therein); (b) Example of the results obtained running (top) a homodimer, Ribonucleoside-diphosphate reductase, PDB id: 3O0N; and (bottom) an heterodimer, the complex of Nuclear pore complex proteins Nup107-Nup133, PDB id: 3CQC. For both cases, a cartoon representation of the proteins with highlighted molecular surfaces in the binding region are depicted on the left. On the right, the corresponding Zernike distances between the 2D projections of the binding region is shown as a vertical line together with the distribution of Zernike distances of a set of 100 decoys. 2D projections of the binding patches are represented as color maps above the distributions (see main text for details). (c) Area under the curve of the ROC as a function of the order of expansion and patch radius for the 4600 analyzed complexes.

The Zepyros web application allows assessing the shape complementarity at the binding region of a given protein–protein complex from a starting structure file in either PDB, CIF, or MMCIF formats (see Fig. 1a). The tool is based on the following steps: (i) the user is required to select two interacting partners, for which the solvent-exposed molecular surfaces are evaluated. Binding regions are identified as the set of residues having surface points closer than 3Å. (ii) The identified binding patches are rotated in such a way that the mean normal versor of the template patch is oriented toward the positive *z*-axis, while the one of the target patch is oriented along the negative *z*-axis. (iii) Both patches are projected into a discretized plane so that each pixel of the plane is weighted according to the distance between the patch points and the origin of a cone containing the patch. (iv) Projected maps (f(r,θ)) are decomposed into the Zernike 2D basis set as $f(r,\theta )=\sum_{n,m}c_{n}^{m}Z_{n}^{m}(r,\theta )$ where $c_{n}^{m}$ are the Zernike complex coefficients and $Z_{n}^{m}$ the Zernike basis polynomials. (v) Given two patches, their complementarity can be measured evaluating the Euclidean distance between the squared modulus of the corresponding two sets of coefficients (where the modulus ensure rotational invariance to the resulting descriptors): $D_{z}=\sqrt{\sum_{n,m}{\left(|c_{n}^{m}|-|c^{\prime }{}_{n}^{m}|\right)}^{2}}$. Analyzing a large dataset of protein–protein interactions (Milanetti *et al.* 2021), we observed that the binding region is characterized on average by a higher-than-random shape complementarity (see Fig. 1b). In particular, comparing the distribution of real Zernike distances with that obtained between couples of random patches, we found that the two distributions are statistically different as measured by the value of AUC of the ROC curve. In Fig. 1c, we reported the AUC values upon varying the two parameters that the user can set before submitting the job, i.e. the radius of the patches and the order of the expansion. One can see that while the AUC is always higher than 0.5, the highest scores are obtained for a patch of ∼8Å and an order of expansion greater than 10. Note that using an expansion order higher than 20 does not further increase the method performance.

## 3 Input and output description

### 3.1 Input

Following the link: https://zepyros.bio-groups.com, the user reaches the main page (Fig. 1a), where she/he can upload the structure of the protein–protein complex of interest using the upload button. The size of the provided structure file must not exceed 10 MB due to memory issues. As sample data, we discuss in the tutorial the case of ribonucleoside-diphosphate reductase (PDB id: 3O0N).

### 3.2 Output

The output of a Zepyros run consists of: (i) The Zernike distance between the two interacting patches, providing a measure of the shape complementarity at the interface. While lower the distance, the higher the complementarity, we provide a reference frame, comparing the distance with the distribution of distances obtained confronting the target patch with a set of random decoys sampled on the target surface. (ii) The graphical representation of the two real patches projected into the xy-plane and confined in the unitary circle (displayed in Fig. 1b). (iii) The values of all the computed Zernike coefficients. All data and figures are available in a zip file, which can be downloaded.

### 3.3 Documentation/tutorials

Zepyros has a user-friendly interface that guides how to use the tool and interpret the results. The documentation page contains a standalone in-depth guide discussing the data submission, as well as of the method and the exploration of the results with case examples (two tutorials are provided). It can be found at: https://zepyros.bio-groups.com/documentation

### 3.4 System requirements

The web server requires the most recent version of the following browsers with JavaScript enabled: Chrome, Firefox, and Safari. If your browser connects through a proxy, please be aware that you might experience a slow upload of the data in the query forms.

## 4 Conclusion

The Zepyros tool is rapid and able to provide results for two average proteins in ∼3 min (depending on the selected number of random patches to be evaluated), while for larger complexes, the waiting time is of nearly 5 min. Moreover, the webserver is user-friendly and can be run without any *a priori* knowledge on theoretical or computational biology. We believe the measure of shape complementarity provided by the Zepyros server could be of use in a number of practical applications.

## Data Availability

Authors confirm that all relevant data are included in the paper.
